# ONX-0914 Alleviates Impaired Diabetic Wound Healing by Restoring Redox Homeostasis and Modulating Pro-Inflammatory Response

**DOI:** 10.3390/medicina62061122

**Published:** 2026-06-09

**Authors:** Betül Çıkı, Damla Kayalı, Hafize Uzun, Necdet Altıner, Abdulhalim Şenyiğit, Betül Yılmaz

**Affiliations:** 1 Department of Medical Biochemistry, Faculty of Medicine, Marmara University, Istanbul 34854, Türkiye; betul.ciki@atlas.edu.tr (B.Ç.); betulkarademir@marmara.edu.tr (B.Y.); 2 Department of Biochemistry, Faculty of Medicine, Istanbul Atlas University, Istanbul 34408, Türkiye; 3 Department of Histology and Embryology, Faculty of Medicine, Marmara University, Istanbul 34854, Türkiye; damlagokceoglu@yandex.com; 4 Department of Histology and Embryology, Faculty of Medicine, Maltepe University, Istanbul 34854, Türkiye; necdetaltiner@maltepe.edu.tr; 5 Department of Internal Medicine, Faculty of Medicine, Istanbul Atlas University, Istanbul 34408, Türkiye; abdulhalim.senyigit@atlas.edu.tr

**Keywords:** diabetic wound healing, ONX-0914, immunoproteasome inhibition, oxidative stress, redox homeostasis, inflammation, streptozotocin-induced diabetes, antioxidant enzymes, cytokines, rat model

## Abstract

*Background and Objectives*: Diabetes mellitus (DM) is frequently associated with impaired wound healing due to persistent oxidative stress, chronic inflammation, and dysregulated proteasome activity, leading to delayed tissue repair and increased risk of chronic ulcers. The present study aimed to investigate the role of the immunoproteasome system in diabetic wound healing, with a particular focus on its involvement in oxidative stress and inflammatory pathways, and to evaluate whether pharmacological inhibition with ONX-0914 improves tissue repair. *Materials and Methods*: Experimental diabetes was induced in rats using streptozotocin (STZ), and the animals were allocated to three groups: healthy control, STZ-induced diabetic, and STZ + ONX-0914 treatment. Wound healing was evaluated by macroscopic analysis of wound closure and histopathological examination at days 3, 7, and 14. Oxidative stress and antioxidant status were assessed by measuring malondialdehyde (MDA) levels and antioxidant enzyme activities (SOD, CAT, and GSH-Px) in serum and wound tissues. Proteasome activity was analyzed fluorometrically, while systemic and local inflammatory responses were determined by ELISA and Western blot analysis of IL-1β, TNF-α, and IL-6. *Results*: STZ-induced diabetes significantly delayed wound closure, increased lipid peroxidation, reduced antioxidant enzyme activities, and elevated systemic and tissue inflammatory cytokine levels. Treatment with ONX-0914 markedly accelerated wound closure and improved histological healing parameters, including re-epithelialization, granulation tissue formation, and angiogenesis. Moreover, ONX-0914 significantly reduced MDA levels while restoring SOD, CAT, and GSH-Px activities in both serum and wound tissues. The treatment also inhibited proteasome activity and significantly suppressed the expression of IL-1β, TNF-α, and IL-6. *Conclusions*: ONX-0914 significantly improves diabetic wound healing by restoring antioxidant defenses, reducing oxidative damage, and attenuating inflammatory signaling pathways. These findings suggest that immunoproteasome inhibition represents a promising therapeutic strategy for enhancing tissue repair in diabetic conditions.

## 1. Introduction

Diabetes mellitus (DM) is a clinically and epidemiologically heterogeneous metabolic disorder encompassing type 1 (T1DM), type 2 (T2DM), gestational, and rare monogenic forms. Its global burden has increased markedly, with an adult prevalence of approximately 11%, of which 90–95% corresponds to T2DM. Although T1DM accounts for a smaller proportion, its incidence and absolute number continue to rise, particularly among children and adolescents. Between 1990 and 2021, the age-standardized prevalence increased by ~19.5% for T1DM and ~118% for T2DM, largely driven by aging, urbanization, obesity, physical inactivity, and Western dietary patterns [1,2,3].

Diabetic foot ulcers (DFUs), affecting approximately 7% of individuals with diabetes, are a major cause of morbidity and hospitalization [4]. These chronic wounds arise from a multifactorial interplay of peripheral neuropathy, ischemia, vascular insufficiency, and biomechanical stress, and may progress to deeper tissue involvement [4,5]. Impaired healing in DFUs is characterized by persistent inflammation, excessive reactive oxygen species (ROS) production, delayed re-epithelialization, and sustained pro-inflammatory cytokine expression [6,7]. In addition, hyperglycemia-induced immune dysfunction and microvascular damage further exacerbate chronicity and defective tissue repair [8]. Recent therapeutic paradigms emphasize modulating local immune cells and their intracellular proteolytic regulators to suppress the chronic inflammatory cascade [9,10]. In this context, precisely delineating the immunoproteasome-driven pathways that perpetuate excessive cytokine production within the pathological wound microenvironment is critical. Despite their clinical burden, the molecular mechanisms underlying diabetic wound healing remain incompletely understood.

In the microenvironment of chronic diabetic wounds, sustained hyperglycemia is a primary driver of the overproduction of reactive oxygen species (ROS), which rapidly overwhelms endogenous antioxidant defenses [11]. This state of persistent oxidative stress does not function in isolation; rather, it feeds into a continuous pathogenic loop with chronic, unresolved inflammation [12]. Emerging evidence indicates that this hyperinflammatory baseline strongly upregulates immunoproteasome expression—a specialized multi-catalytic protease complex—which is predominantly active in immune cells under stress conditions [13]. While the constitutive proteasome handles routine cellular housekeeping, the immunoproteasome rapidly processes pro-inflammatory signaling intermediates, thereby driving upstream translational and post-translational processing of key inflammatory mediators. This molecular activation triggers an unregulated transcriptional surge of canonical pro-inflammatory cytokines, specifically interleukin-1β (IL-1β), tumor necrosis factor-α (TNF-α), and interleukin-6 (IL-6) [14,15]. Consequently, the wound bed becomes frozen in a permanent inflammatory phase, actively preventing the essential transition into the proliferative and re-epithelialization stages of tissue repair [16].

ONX-0914 (PR-957) is a selective immunoproteasome inhibitor targeting the LMP7 (β5i) and LMP2 (β1i) subunits, exhibiting potent immunomodulatory effects with reduced toxicity compared to conventional proteasome inhibitors [15]. It has demonstrated therapeutic efficacy in multiple disease models by suppressing inflammatory pathways and cytokine production [15,16], with an improved specificity and safety profile compared with agents such as bortezomib and carfilzomib [17,18,19,20].

The present study aimed to investigate the therapeutic potential of the selective immunoproteasome inhibitor ONX-0914 in diabetic wound healing, with a particular focus on its effects on oxidative stress, proteasome activity, and inflammatory responses. Using a streptozotocin (STZ)-induced diabetic rat wound model, we evaluated whether immunoproteasome inhibition could improve wound repair by modulating both systemic and local pathological processes.

Although general proteasome dynamics have been investigated in various inflammatory conditions [13,16], how targeted immunoproteasome modulation influences the chronological stages of diabetic wound healing remains uncharted. The current investigation advances beyond existing publications by providing a precise, multi-methodological time-course analysis of immunoproteasome inhibition via ONX-0914, explicitly demonstrating its downstream effects in restoring redox balance and orchestrating the pro-inflammatory microenvironment to rescue delayed epithelial repair.

We hypothesized that ONX-0914 treatment would accelerate wound healing in diabetic conditions by restoring redox homeostasis, enhancing antioxidant defense mechanisms, and suppressing excessive inflammatory responses through selective inhibition of immunoproteasome activity. Furthermore, we postulated that these effects would be associated with reduced lipid peroxidation, decreased pro-inflammatory cytokine expression, and improved histopathological healing outcomes.

## 2. Materials and Methods

### 2.1. Animals and Ethical Approval

A total of 28 adult male Wistar rats (10–12 weeks old, weighing 250–300 g) were used in this study. The animals were housed in a controlled environment (18–24 °C, 50–60% humidity) with a 12 h light/dark cycle and provided with ad libitum access to standard rodent chow and water. All experimental procedures were approved by the Maltepe University Animal Ethics Committee (MÜHADYEK, Permit No: [2023.08.02]) and conducted in accordance with the NIH Guide for the Care and Use of Laboratory Animals. To prevent mechanical stress, scratching, or autotomy of the healing tissue, excisional wounds were strategically created in the dorsal interscapular region of each rat, an anatomical site inaccessible to the animals. Post-operatively, rats were housed individually under strictly controlled environmental conditions (22 ± 2 °C, 50–60% humidity) with daily cage sanitization to maintain hydration and reduce the risk of infection.

### 2.2. Induction of Diabetes and Wound Model

Diabetes was induced by a single intraperitoneal injection of streptozotocin (STZ, 60 mg/kg, Cayman, Ann Arbor, MI, USA) dissolved in citrate buffer (pH 4.5). Fasting blood glucose (FBG) was measured 72 h post-injection via a tail vein glucometer; only rats with FBG ≥ 250 mg/dL were considered diabetic.

Following anesthesia with ketamine (25 mg/kg) and xylazine (10 mg/kg), the dorsal area was shaved and disinfected. Two full-thickness excisional wounds (1 cm × 1 cm) were created on the mid-back of each rat using a sterile biopsy punch. The animals were randomly assigned to three groups: (1) healthy control, untreated non-diabetic wound (*n* = 4), (2) STZ-induced diabetic group (*n* = 12), and (3) ONX-0914-treated diabetic group (*n* = 12). The sample size for the experimental groups was determined a priori using G*Power software (version 3.1.9.7). In this treatment cohort, the inhibitor (10 mg/kg, Cayman) was administered via subcutaneous (S.C.) injection at a volume of 100–150 µL per injection. This agent was dissolved in 5% DMSO and further diluted in sterile saline (0.9% NaCl) prior to administration. The treatment was performed three times weekly throughout the experimental period. To ensure experimental consistency, control and STZ-only groups received an equivalent volume of the vehicle (5% DMSO in saline) via the same route. Preliminary evaluations and chronological comparisons between untreated controls and vehicle-treated groups revealed no macroscopic or microscopic toxicity, localized necrosis, or systemic interference in the diabetic wound-healing continuum.

### 2.3. Histopathological Evaluation and Wound Closure Analysis

The wound healing process was monitored by capturing digital photographs of the wound sites on days 3, 7, and 14. The wound area was quantified using ImageJ software (v1.54k) (National Institutes of Health, Bethesda, MD, USA) by tracing the wound margins. The wound closure rate was calculated as a percentage of the initial wound area using the following formula:

Wound Closure (%) = [(Area on Day 0 − Area on Day *n*)/Area on Day 0] × 100.

For histopathological assessment, wound tissue samples collected on days 3, 7, and 14 were fixed in 10% buffered formalin, dehydrated, and embedded in paraffin. Sections (5 μm thickness) were stained with Hematoxylin and Eosin (H&E). A semi-quantitative scoring system was utilized to evaluate re-epithelialization, granulation tissue formation, inflammatory cell infiltration, and angiogenesis. The detailed grading criteria and weightings are summarized in Table 1. The sections were examined and photographed under a light microscope by a pathologist blinded to the experimental groups.

Wound tissue sections were stained with Masson’s Trichrome staining to evaluate collagen deposition during the wound healing process. Histological images were captured under a light microscope at the same magnification for all groups. Quantitative analysis of collagen deposition was performed using ImageJ software (v1.54k) (National Institutes of Health, Bethesda, MD, USA). The blue-stained collagen-positive areas were selected using color thresholding, and the percentage of collagen deposition was calculated as the ratio of the blue-stained area to the total tissue area. The results were expressed as a percentage (%) of collagen-positive area for each experimental group and time point.

### 2.4. Serum and Tissue Biochemical Analyses

Serum and tissue samples were analyzed to evaluate systemic and local biochemical changes. Serum concentrations of pro-inflammatory cytokines, including Tumor Necrosis Factor-alpha (TNF-α, Catalog # MBS2507393), Interleukin-1 beta (IL-1β, Catalog # MBS825017), and Interleukin-6 (IL-6, Catalog # MBS269892), were quantified using rat-specific ELISA kits (MyBioSource, San Diego, CA, USA) according to the manufacturer’s instructions. The absorbance was measured at 450 nm using a microplate reader (PerkinElmer, Waltham, MA, USA).

For oxidative stress assessment, lipid peroxidation was determined by measuring Malondialdehyde (MDA, Cat: E0156Ra) levels in both serum and tissue lysates. Additionally, the activities of key antioxidant enzymes, namely Superoxide Dismutase (SOD, Cat: E0168Ra), Catalase (CAT, Cat: E0869Ra), and Glutathione Peroxidase (GSH-Px, Cat: E1172Ra), were measured using colorimetric assays (Bioassay Technology Laboratory, Jiaxing, China). To ensure accurate comparison of enzyme activities in tissue samples, protein concentrations in the lysates were standardized using the Pierce™ BCA Protein Assay Kit (Thermo Scientific, Rockford, IL, USA). All absorbance expression of the immunoproteasome -a specialized multi-catalytic protease complex- predominantly active and colorimetric measurements were performed using the EnSpire™ Multimode Plate Reader (PerkinElmer, Waltham, MA, USA).

### 2.5. Measurement of Proteasome Activity

Immunoproteasome chymotrypsin-like activity was measured using the fluorogenic substrate Suc-LLVY-AMC, a method widely validated in studies investigating the selective inhibitory effects of ONX-0914 on the LMP7 subunit [15]. Briefly, tissue samples were homogenized in a lysis buffer without proteasome inhibitors. The protein concentration was standardized using the BCA assay (Pierce™ BCA Protein Assay Kit, Thermo Scientific, Rockford, IL, USA). The lysates were incubated with the substrate, and the release of free AMC was monitored periodically using a fluorescence plate reader at 380 nm excitation and 460 nm emission (PerkinElmer, Waltham, MA, USA). Proteasome activity was expressed as relative fluorescence units (RFU) per microgram of protein.

### 2.6. Western Blot Analysis

Total protein was extracted from the collected wound tissues. Briefly, tissue samples were placed in tubes containing an appropriate volume of RIPA lysis buffer supplemented with protease and phosphatase inhibitors. For efficient tissue disruption, Zirconium Oxide Beads (1.0 mm diameter, Next Advance (Troy, NY, USA) were added to each tube, and the samples were homogenized using a bead mill homogenizer (Next Advance, Troy, NY, USA) until a uniform homogenate was obtained.

The homogenates were then centrifuged at 4 °C, and the supernatants were collected. Protein concentrations were determined using the BCA protein assay kit (Pierce™, Thermo Scientific). Equal amounts of protein (30 μg) were separated by SDS-PAGE and transferred onto PVDF membranes. The membranes were blocked with 5% non-fat dry milk for 1 h at room temperature and then incubated overnight at 4 °C with the primary antibodies, purchased from Elabscience Biotechnology Inc. (Houston, TX, USA), included TNF-α (1:1000), IL-1β (1:1000), and IL-6 (1:1000). GAPDH was used as an internal loading control to ensure equal protein loading (1:1000, Cell Signaling, Danvers, MA, USA). For sequential protein detection on the same blots, a stripping-and-reprobing protocol was employed. Following IL-6 visualization, the membranes were incubated in a commercial stripping buffer (Catalog # 21059, Thermo Scientific, Waltham, MA, USA) at 50 °C for 30 min to remove bound antibodies (see Supplementary Materials). After incubation with HRP-conjugated secondary antibodies (1:10,000, Catalog # 31460, Thermo Scientific, Waltham, MA, USA), protein bands were visualized using an enhanced chemiluminescence (ECL) kit (Pierce™, Thermo Scientific, Waltham, MA, USA) and quantified via densitometric analysis using ImageLab software (v6.1) (Bio-Rad Laboratories, Hercules, CA, USA).

### 2.7. Statistical Analysis

Data are expressed as mean ± standard error of the mean (SEM). Statistical comparisons were performed using GraphPad Prism 10.0 software (Boston, MA, USA). Differences between groups were analyzed by two-way ANOVA followed by Tukey’s or Bonferroni post hoc tests for multiple comparisons. A *p*-value of <0.05 was considered statistically significant.

## 3. Results

### 3.1. Validation of the STZ-Induced Diabetes Model

To establish the experimental diabetic model, rats were treated with a single dose of streptozotocin (STZ). Fasting blood glucose (FBG) levels were measured 72 h post-injection to confirm hyperglycemia. As shown in Table 2, the FBG levels of the Control group remained within the physiological range (98 ± 12 mg/dL). In contrast, rats in the STZ and STZ + ONX-0914 groups exhibited significantly elevated glucose levels (294 ± 18 mg/dL and 287 ± 11 mg/dL, respectively), exceeding the diabetic threshold of 250 mg/dL. No significant difference in FBG levels was observed between the two diabetic groups at the beginning of the treatment period, ensuring a uniform baseline for assessing the effects of ONX-0914 on wound healing.

### 3.2. Effects of ONX-0914 on Macroscopic Wound Closure and Histopathological Recovery

**Figure 1:**
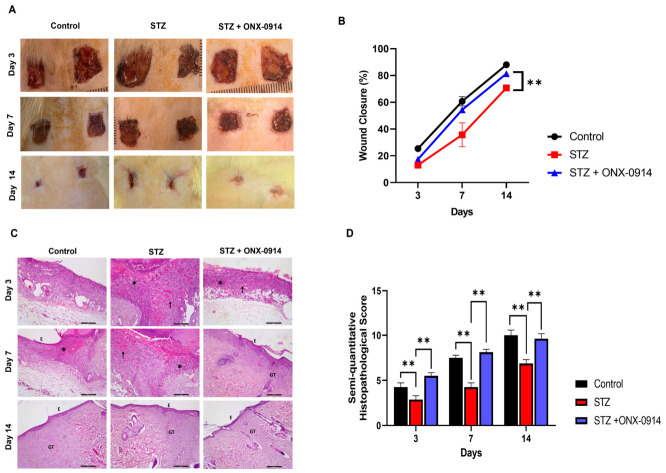
Time-dependent effect of ONX-0914 on wound healing rates. (**A**) Representative macroscopic images of wounds at Day 3, 7, and 14. (**B**) Quantitative analysis of wound closure rate (%). (**C**) Histopathological evaluations. Representative hematoxylin and eosin (H&E) stained wound sections obtained from control, STZ and STZ + ONX-0914 groups on days 3, 7 and 14. * inflammatory cell infiltration; E, epithelium; GT, granulation tissue; arrows indicate neovascularization. Scale bars: 200 µm. (**D**) Graphical representation of total histological scores based on re-epithelialization, granulation tissue formation, inflammatory cell infiltration, and angiogenesis. Data are presented as mean ± SEM (*n* = 4). Statistical significance was determined by two-way ANOVA followed by Tukey’s post hoc test. ** *p* < 0.01 vs. Control and STZ group.

The wound healing process was significantly delayed in the STZ-induced diabetic group compared to the healthy control group. Macroscopic observations revealed that the STZ group had larger wound areas on days 7 and 14 (Figure 1). However, treatment with ONX-0914 significantly accelerated the wound closure rate, particularly from day 7 onwards, reaching levels comparable to the control group by day 14.

Histopathological analysis via H&E staining supported these findings. In the STZ group, persistent inflammatory cell infiltration, reduced granulation tissue formation, and delayed re-epithelialization were observed (Table 3). Conversely, the ONX-0914-treated group exhibited a marked reduction in inflammatory cell density and improved dermal structural organization, characterized by enhanced angiogenesis and a more robust epithelial covering (Figure 1). Semi-quantitative histological scoring confirmed that ONX-0914 significantly improved total healing scores compared to the untreated diabetic group (** *p* < 0.01).

The raw individual total scores for each animal across all experimental groups and time points (Days 3, 7, and 14) are presented in Table 3 to demonstrate the consistency of our histopathological findings. This individual dataset directly supports and validates the mean total histological scores illustrated in Figure 1D.

**Figure 2:**
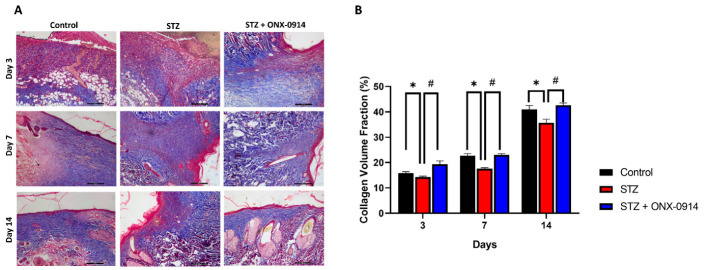
Chronological evaluation of collagen deposition in diabetic wound healing. (**A**) Representative light microscopy images of wound sections stained with Masson’s Trichrome at Days 3, 7, and 14 post-injury across the Control, STZ, and STZ + ONX-0914 experimental groups (Scale bars = 200 µm). (**B**) Statistical quantification of the Collagen Volume Fraction (%) measured from the histological sections across the respective time points (Days 3, 7, and 14). Data are presented as mean ± SEM (*n* = 4). * *p* < 0.05 indicates a statistically significant difference compared to the Control group; # *p* < 0.05 indicates a statistically significant difference compared to the untreated STZ group.

Temporal changes in collagen deposition were monitored via Masson’s Trichrome staining to assess the structural quality of the extracellular matrix during tissue repair (Figure 2A). In the untreated STZ group, systemic diabetic stress and persistent inflammation significantly impaired matrix synthesis, resulting in a sparse, fragmented, and highly disorganized collagen network throughout the healing timeline. This structural deficiency directly correlated with the delayed wound closure.

Conversely, selective immunoproteasome inhibition via ONX-0914 markedly rescued this tissue impairment. The STZ + ONX-0914 group demonstrated a robust and statistically significant increase in Collagen Volume Fraction (%) compared to the untreated STZ group by Day 14 (Figure 2B). Visually, this therapeutic intervention accelerated the formation of dense, well-organized, and mature collagen bundles, confirming that ONX-0914 effectively drives the diabetic wound microenvironment into a functional fibroblastic remodeling phase.

### 3.3. Mitigation of Systemic and Local Oxidative Stress

The impact of ONX-0914 on the redox status was evaluated by analyzing lipid peroxidation and antioxidant enzyme activities in both systemic circulation and local wound tissues.

**Figure 3:**
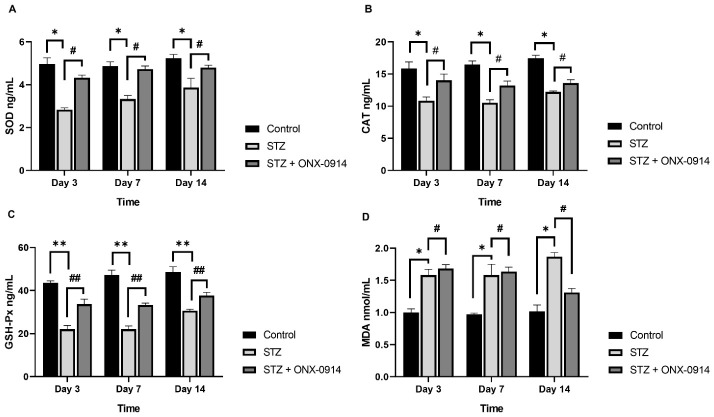
ONX-0914 restores antioxidant balance and reduces lipid peroxidation in diabetic rats. Serum levels of (**A**) SOD, (**B**) CAT, (**C**) GSH-Px, and (**D**) MDA were analyzed across Day 3, 7, and 14. STZ-induced diabetes led to a significant depletion of antioxidant enzyme activities (SOD, CAT, GSH-Px) and a concomitant rise in lipid peroxidation (MDA) levels. Administration of the immunoproteasome inhibitor ONX-0914 effectively reversed these pathological changes by significantly increasing antioxidant defenses and suppressing oxidative damage throughout the experimental period. Data are expressed as mean ± SEM. Statistical significance was determined by two-way ANOVA followed by a Bonferroni post hoc test. * *p* < 0.5 vs. Control group; ** *p* < 0.01 vs. Control group; # *p* < 0.05 vs. STZ group; ## *p* < 0.01 vs. STZ group at the respective time point.

Systemic redox profiling revealed that STZ-induced diabetes triggered significant oxidative stress throughout the body. Serum MDA (malondialdehyde) levels were markedly higher in the STZ group compared to healthy controls, indicating widespread lipid peroxidation. Conversely, treatment with ONX-0914 effectively attenuated this systemic oxidative stress, as evidenced by a significant reduction in serum MDA levels. Furthermore, the activities of key systemic antioxidant enzymes SOD, CAT, and GSH-Px, which were severely depleted in diabetic rats, were significantly restored following ONX-0914 administration. This systemic recovery suggests that immunoproteasome inhibition strengthens the overall antioxidant defense mechanism (Figure 3).

**Figure 4:**
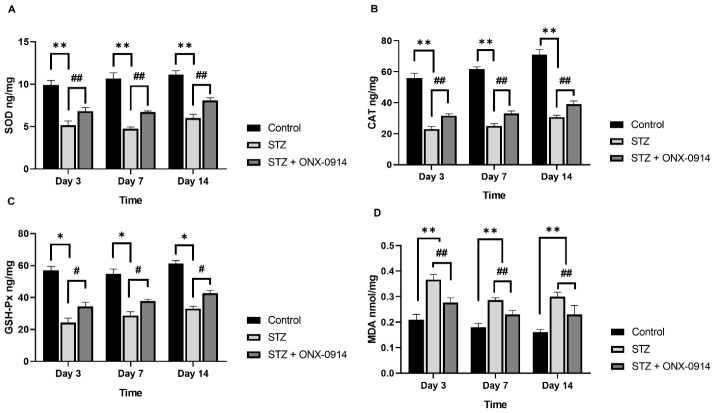
ONX-0914 attenuates oxidative stress in tissues and enhances antioxidant capacity in diabetic rats. Tissue levels of (**A**) SOD, (**B**) CAT, (**C**) GSH-Px, and (**D**) MDA were analyzed in control, STZ-induced diabetic, and ONX-0914-treated diabetic groups. STZ administration caused a profound reduction in tissue antioxidant enzyme activities (SOD, CAT, GSH-Px) and a significant increase in MDA levels, indicating severe lipid peroxidation. Treatment with ONX-0914 significantly restored the antioxidant enzyme profile and successfully suppressed tissue oxidative damage across all time points (Day 3, 7, and 14). Data are expressed as mean ± SEM (*n* = 4 per group). Statistical significance was determined by two-way ANOVA followed by a Bonferroni post hoc test. * *p* < 0.5 vs. Control group; ** *p* < 0.01 vs. Control group; # *p* < 0.05 vs. STZ group; ## *p* < 0.01 vs. STZ group at the respective time point.

Consistent with these systemic improvements, the local redox environment at the wound site showed a similar recovery pattern. In the wound lysates of the STZ group, MDA levels were significantly elevated, reflecting severe local tissue damage. However, ONX-0914 treatment markedly reduced local MDA concentrations, particularly during the critical healing phases on days 7 and 14. This was accompanied by a significant restoration of local SOD, CAT, and GSH-Px activities. To ensure the technical validity of these tissue-based findings, GAPDH was used as the internal loading control for protein normalization, confirming that the enhancement of the antioxidant capacity was specific to the treatment (Figure 4).

Taken together, these results demonstrate that ONX-0914 provides a dual-action therapeutic effect by first alleviating systemic oxidative burden and subsequently restoring the local antioxidant balance, thereby creating a favorable environment for accelerated wound repair in diabetic conditions.

### 3.4. Inhibition of Proteasome Activity and Modulation of Systemic Inflammation

**Figure 5:**
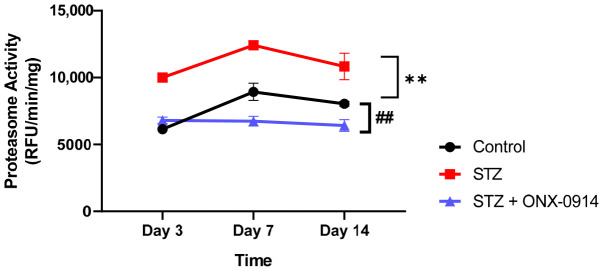
Fluorometric proteasome activity assay. Statistical significance between groups was determined by two-way ANOVA. Data are expressed as mean ± SEM (*n* = 4 per group). ** *p* < 0.01 vs. Control group; ## *p* < 0.01 vs. STZ group at the respective time points.

To confirm the efficacy of ONX-0914, we measured proteasome activity in skin tissues. STZ induction led to up-regulation of proteasome activity, whereas ONX-0914 treatment effectively inhibited it at all time points, confirming the inhibitor’s targeted action (Figure 5).

**Figure 6:**
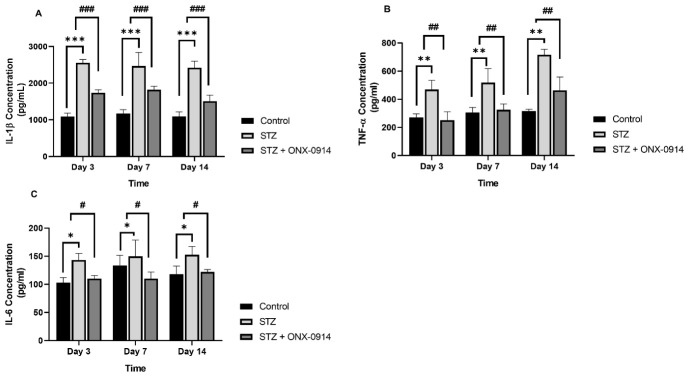
ONX-0914 suppresses systemic pro-inflammatory cytokine levels in diabetic rats. Serum concentrations of (**A**) IL-1β (**B**) TNF-α and (**C**) IL-6 were determined by ELISA on days 3, 7, and 14. Streptozotocin (STZ) induction induced a significant systemic inflammatory response, characterized by sustained elevations in all three cytokines throughout the experimental period. Statistical significance was determined by two-way ANOVA followed by a Bonferroni post hoc test. * *p* < 0.05, ** *p* < 0.01, *** *p* < 0.001 compared to the Control group; # *p* < 0.05, ## *p* < 0.01, ### *p* < 0.001 vs. STZ group at the respective time points. Data are expressed as mean ± SEM (*n* = 4 per group).

Systemic inflammation was assessed by measuring serum levels of pro-inflammatory cytokines. STZ-induced diabetes led to sustained elevations in IL-1β, TNF-α, and IL-6 throughout the 14-day period. ONX-0914 treatment significantly attenuated the serum concentrations of these cytokines (** *p* < 0.01). These systemic changes were mirrored by Western blot analysis of tissue lysates, which showed that ONX-0914 suppressed the intracellular expression of pro-inflammatory markers, thereby limiting the local inflammatory response at the wound site (Figure 6).

### 3.5. ONX-0914 Suppresses Systemic and Local Pro-Inflammatory Response

**Figure 7:**
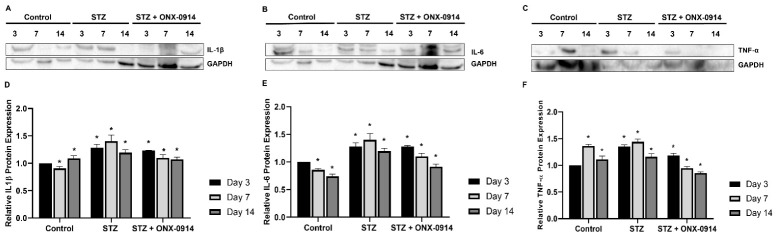
Effect of ONX-0914 on pro-inflammatory cytokine expression. (**A**–**C**) Representative Western blot images and (**D**–**F**) densitometric analysis of pro-IL-1β (31 kDa), IL-6 (28–30 kDa), and pro-TNF-α (26 kDa) in tissue lysates (Day 3, 7, 14). GAPDH served as the loading control. Since mature forms of IL-1β and TNF-α were below the detection limit in tissue lysates, their active levels were confirmed via serum ELISA (Figure 5). STZ-induced up-regulation of both intracellular pro-forms and circulating mature cytokines was significantly attenuated by ONX-0914 treatment across all time points. Data are mean ± SEM (*n* = 4). Two-way ANOVA, * *p* < 0.05 vs. Control Day 3.

Complementary to the systemic findings, Western blot analysis of wound tissue lysates was performed to evaluate the local expression of pro-inflammatory markers, with GAPDH as the internal loading control to ensure equal protein loading across all samples. STZ induction significantly up-regulated the intracellular expression of pro-IL-1β, pro-TNF-α, and IL-6 (Figure 7). Densitometric analysis of the bar graphs showed that ONX-0914 treatment effectively suppressed the protein expression of these markers at all time points. While the mature forms of IL-1β and TNF-α were primarily detectable in the serum, the significant reduction in their intracellular precursor levels in the ONX-0914 group, normalized to GAPDH, confirms that immunoproteasome inhibition targets the inflammatory cascade at its source. These results demonstrate that ONX-0914 provides a dual-layer anti-inflammatory effect by modulating both local tissue expression and systemic circulation of key cytokines.

Proteasome chymotrypsin-like activity in wound tissue lysates was quantified by measuring the fluorescence of released AMC. The raw data, expressed as Relative Fluorescence Units (RFU), were normalized to the total protein content of each sample (RFU/µg protein). A significant reduction in RFU values was observed in the ONX-0914-treated group compared with the diabetic control group, confirming effective inhibition of the immunoproteasome at the wound site.

## 4. Discussion

The molecular pathogenesis of delayed diabetic wound healing represents a complex clinical challenge, long characterized by a self-perpetuating cycle of severe oxidative stress and chronic, unresolved inflammation [11,14]. The current study provides the first comprehensive, time-course evidence demonstrating that the selective immunoproteasome inhibitor ONX-0914 is a potent therapeutic agent that breaks this pathogenic continuum, independent of systemic glycemic modulation. By demonstrating that targeted β5i subunit inhibition simultaneously restores tissue redox homeostasis and suppresses systemic and local pro-inflammatory cytokine cascades [13], our findings offer a novel mechanistic paradigm for rescuing compromised dermal tissue repair under diabetic stress.

The validity of our experimental model was confirmed by sustained hyperglycemia following STZ administration, with comparable baseline glucose levels between the diabetic cohorts. This uniform baseline is critical, as it excludes confounding glycemic differences and confirms that the observed therapeutic benefits are directly attributable to ONX-0914. Chronic hyperglycemia is well known to impair wound healing by promoting oxidative damage, prolonged inflammation, and defective angiogenesis, ultimately disrupting systemic and localized tissue repair processes [21,22,23]. In line with these mechanisms, untreated diabetic wounds in this study exhibited severely delayed closure and impaired histological recovery, characterized by persistent inflammatory cell infiltration, reduced granulation tissue formation, and delayed re-epithelialization.

ONX-0914 treatment markedly improved these parameters, accelerating macroscopic wound closure and enhancing key histopathological features. To evaluate the structural quality of tissue repair, the temporal dynamics of collagen architecture were monitored via Masson’s Trichrome staining. Collagen deposition represents a fundamental cornerstone of functional wound healing, providing the essential extracellular matrix (ECM) scaffolding required for efficient re-epithelialization [24]. In the untreated STZ group, persistent inflammation and diabetic stress resulted in a sparse, fragmented, and highly disorganized collagen network, directly accounting for the defective wound closure. Conversely, selective immunoproteasome inhibition with ONX-0914 significantly rescued this matrix impairment, resulting in a robust increase in dense, well-organized, and mature collagen bundles by Day 14. This structural enhancement confirms that ONX-0914 successfully transitions the diabetic wound microenvironment from a chronic inflammatory phase into a functional fibroblastic remodeling stage [14,25,26].

Oxidative stress is a central pathological driver in diabetic wounds, where excessive reactive oxygen species (ROS) production and depleted endogenous antioxidant defenses disrupt cellular viability and tissue regeneration [11,27,28]. Hyperglycemia-induced mitochondrial dysfunction and subsequent activation of oxidative pathways impair angiogenesis, matrix remodeling, and localized cellular proliferation [13,29]. In our model, STZ-induced diabetes triggered profound lipid peroxidation—evidenced by elevated malondialdehyde (MDA) levels—alongside a severe depletion of key antioxidant enzymes (SOD, CAT, and GSH-Px) in both serum and wound tissues [25,27,30]. ONX-0914 treatment effectively reversed these alterations at both systemic and local levels, demonstrating its potent efficacy in restoring redox homeostasis [23,31].

Mechanistically, the significant reduction in MDA levels following ONX-0914 administration indicates robust suppression of oxidative membrane degradation, thereby preserving the structural integrity of cells, which is vital for keratinocyte and fibroblast migration. In diabetes, elevated MDA levels strongly correlate with heightened pro-inflammatory signaling, particularly via the NF-κB pathway. Emerging literature suggests that immunoproteasome inhibition via ONX-0914 reduces the proteolytic processing of the p105 precursor into active p50 subunits, thereby dampening the inflammatory surge that drives oxidative tissue damage [23,32]. Furthermore, the dramatic restoration of SOD, CAT, and GSH-Px activities underscores the dual-action therapeutic profile of ONX-0914. While SOD manages the initial dismutation of superoxide radicals, CAT and GSH-Px are indispensable for detoxifying hydrogen peroxide (H_2_O_2_) into water, thereby interrupting the formation of highly destructive hydroxyl radicals via the Fenton reaction. The recovery of these enzymatic activities suggests that immunoproteasome inhibition may stabilize critical antioxidant transcription factors, potentially preventing the proteasomal degradation of Nrf2 (nuclear factor erythroid 2-related factor 2), a master regulator of the antioxidant response element (ARE) under high-glucose stress [33,34].

Dysregulation of proteasome activity represents a critical but frequently underexplored axis in chronic wound pathology. In the present study, STZ-induced diabetes was associated with a marked upregulation of proteasome chymotrypsin-like activity in wound tissue, which was effectively and selectively suppressed by administration of ONX-0914. Emerging evidence indicates that immunoproteasome activation is inextricably linked to hyper-inflammatory signaling cascades, sustaining chronicity and hindering tissue resolution [35].

Regarding systemic inflammation, our diabetic animals exhibited pronounced elevations in circulating IL-1β, TNF-α, and IL-6, which are well-established mediators of chronic inflammation and tissue impairment. At the tissue level, Western blot analyses corroborated these systemic findings, demonstrating that STZ-induced diabetes markedly increased the localized expression of pro-IL-1β, pro-TNF-α, and IL-6, thereby confirming the persistent activation of local inflammatory pathways. IL-1β is notorious for prolonging the inflammatory phase of wound healing and disrupting keratinocyte migration, thereby stalling re-epithelialization [36,37]. The observed reduction in pro-IL-1β expression following ONX-0914 treatment strongly suggests that immunoproteasome inhibition limits early inflammatory signaling directly at its upstream molecular source, thereby facilitating a timely transition into the proliferative phase. Concurrently, excessive TNF-α levels drive local endothelial dysfunction and suppress fibroblast proliferation, thereby directly impairing granulation tissue kinetics [38]. Elevated intracellular pro-TNF-α levels in the untreated STZ group reflect sustained inflammatory activation, whereas their profound suppression by ONX-0914 indicates a direct regulatory effect on cytokine synthesis pathways. This down-regulation is particularly relevant as unmitigated TNF-α signaling is fundamentally associated with delayed wound closure and defective matrix remodeling. Furthermore, IL-6 contributes significantly to maintaining unresolved inflammatory baselines in diabetic conditions [39], and its significant downregulation supports the therapeutic role of ONX-0914 in modulating the pathological wound microenvironment.

Importantly, the concurrent reduction in both circulating mature cytokines and their local intracellular precursors highlights a highly coordinated, dual-layer anti-inflammatory mechanism. Mechanistically, these findings are consistent with recent evidence indicating that the immunoproteasome plays a pivotal role in regulating NF-κB–dependent transcription of pro-inflammatory genes [35,37,40,41,42]. By selectively blocking the β5i subunit, ONX-0914 likely disrupts the proteolytic processing of the p105 precursor into active p50 subunits, thereby attenuating cytokine synthesis at both the transcriptional and post-translational levels and preventing the wound bed from becoming molecularly “frozen” in a permanent inflammatory state [16,42]. Crucially, while research investigating proteasome inhibition in diabetic tissue repair remains highly limited in the existing literature, this study introduces a novel perspective by demonstrating the specific therapeutic potential of ONX-0914. Ultimately, our findings advance the understanding of proteasome dynamics from purely descriptive academic observation to a novel, phase-specific therapeutic strategy, highlighting selective modulation of immunoproteasome subunits as a promising paradigm for rescuing compromised diabetic wounds.

In conclusion, the present investigation establishes the immunoproteasome as a critical upstream molecular orchestrator of diabetic wound pathology. Targeted inhibition via ONX-0914 effectively rescues compromised dermal tissue repair by simultaneously disrupting two fundamental pathogenic mechanisms: severe oxidative stress and chronic inflammation. By successfully lowering lipid peroxidation, restoring endogenous antioxidant defenses (SOD, CAT, GSH-Px), and suppressing the canonical pro-inflammatory cytokine cascade (IL-1β, TNF-α, IL-6), ONX-0914 shifts the pathological wound microenvironment into a functional, regenerative remodeling phase. Crucially, because these structural and biochemical improvements were driven entirely by direct immune and redox modulation without relying on the correction of systemic hyperglycemia, selective immunoproteasome subunit inhibition emerges as a highly promising, independent therapeutic strategy capable of overcoming current translational limitations in diabetic foot ulcer management.

Despite the robust experimental design, several limitations should be acknowledged. First, the study was conducted in an STZ-induced diabetic rat model, which primarily reflects type 1 diabetes and may not fully recapitulate the complex pathophysiology of type 2 diabetes or human diabetic foot ulcers. Second, although key oxidative stress and inflammatory markers were comprehensively evaluated, the study did not directly assess upstream signaling pathways such as NF-κB activation or mitochondrial ROS production, which could further clarify the mechanistic role of the immunoproteasome. Third, the sample size was relatively limited, and longer follow-up periods were not included to evaluate long-term tissue remodeling and scar formation. Fourth, the lack of direct immunophenotyping for leukocyte subsets or macrophage polarization (M1 vs. M2 phenotypes) limits our cellular mapping; although the robust down-regulation of IL-1β, TNF-α, and IL-6 strongly implies a functional shift away from an M1-driven chronic inflammatory state, future investigations utilizing flow cytometry or double-immunofluorescence are required to definitively characterize the cellular targets of ONX-0914 within the diabetic wound microenvironment. Finally, dose–response relationships and potential off-target effects of ONX-0914 were not explored, and safety assessments in non-diabetic or healthy tissues remain to be investigated.

### Future Research

Future studies should focus on validating these findings in clinically relevant models, including type 2 diabetes and large-animal wound models. Mechanistic investigations targeting the immunoproteasome–NF-κB–ROS axis, mitochondrial function, and macrophage polarization would provide deeper insight into the molecular pathways involved. In addition, dose optimization, pharmacokinetics, and long-term safety profiles of ONX-0914 should be systematically evaluated. Combining immunoproteasome inhibition with current standard-of-care therapies, such as growth factors or advanced wound dressings, may also reveal synergistic effects. Ultimately, translational studies and early-phase clinical trials are required to determine the therapeutic potential of ONX-0914 in human diabetic wound management.

In conclusion, the present study demonstrates that selective immunoproteasome inhibition with ONX-0914 effectively improves diabetic wound healing by simultaneously targeting two fundamental pathological processes: oxidative stress and chronic inflammation. By restoring redox homeostasis, enhancing antioxidant defense systems, and suppressing key pro-inflammatory cytokines, ONX-0914 promotes a shift from a persistent inflammatory state toward a regenerative healing environment. Importantly, these effects were achieved independently of glycemic modulation, highlighting the immunoproteasome as a critical upstream regulator of diabetic wound pathology. From a translational perspective, these findings position immunoproteasome inhibition as a promising therapeutic strategy that may overcome current limitations in diabetic wound care. Targeting the immunoproteasome–redox–inflammation axis could represent a novel, clinically relevant approach to improve healing outcomes and reduce complications in patients with diabetes.

## Figures and Tables

**Table 1:** Semi-Quantitative Histopathological Wound Healing Scoring System.

Parameter	Score 0	Score 1	Score 2	Score 3
Re-epithelialization	None	Partial, irregular epithelial migration	Prominent but incomplete epithelialization	Complete and organized epithelial layer
Inflammation	Severe inflammation	Moderate inflammation	Mild inflammation	Minimal/Absent (close to healed tissue)
Granulation and Tissue Formation	None	Poor and disorganized granulation tissue	Moderate, partially organized granulation tissue	Dense and well-organized granulation tissue
Angiogenesis	No new vessel formation	Few new blood vessels	Moderate neovascularization	Dense and well-organized capillary network

**Table 2:** Fasting Blood Glucose Levels in Experimental Groups Following Diabetes Induction.

Group No	Group Name	Animals (*n*)	Experimental Procedure	Blood Glucose (mg/dL)
Group 1	Control (Healthy)	4	Standard Diet + Saline	98 ± 12
Group 2	Diabetes (STZ)	12	STZ Injection	298 ± 18
Group 3	Diabetes + ONX-0914	12	STZ + ONX-0914 Treatment	287 ± 16
TOTAL		28		

**Table 3:** Raw Individual Animal Total Histopathological Scores Across Experimental Groups.

Time Points	Control (*n* = 4)	STZ (*n* = 4 per Time Point)	STZ + ONX-0914 (*n* = 4 per Time Point)
Day 3	5.0/3.0/4.0/5.0	3.0/1.0/2.0/2.0	6.0/7.0/6.0/7.5
Day 7	8.0/8.0/7.0/7.0	4.0/5.0/5.0/3.0	10.0/8.0/9.0/8.0
Day 14	9.0/10.0/11.0/9.0	8.0/6.0/7.0/6.0	11.0/9.0/10.0/9.5

## Data Availability

The original contributions presented in this study are included in the article and Supplementary Materials. Further inquiries can be directed to the corresponding author.

## Supplementary Materials

The following supporting information can be downloaded at: https://www.mdpi.com/article/10.3390/medicina62061122/s1, Figure S1. Full-length Western blot membrane showing intracellular pro-IL-1β (31 kDa) and its matching GAPDH (37 kDa) internal loading control across chronological wound tissue subsets; Figure S2. Full-length Western blot membrane showing IL-6 (28-30 kDa) expression and its matching GAPDH (37 kDa) internal loading control across chronological wound tissue subsets; Figure S3. Full-length Western blot membrane showing intracellular pro-TNF-α (26 kDa) and its matching GAPDH (37 kDa) internal loading control across chronological wound tissue subsets.
