# Supplementary material for: ONX-0914 Alleviates Impaired Diabetic Wound Healing by Restoring Redox Homeostasis and Modulating Pro-Inflammatory Response
Source: Medicina (Kaunas). 2026 Jun 9;62(6):1122. doi: 10.3390/medicina62061122 (PMC13304357; doi:10.3390/medicina62061122)
Supplement: Supplementary file 1 [file medicina-62-01122-s001.zip › medicina-4262315-supplementary.pdf]

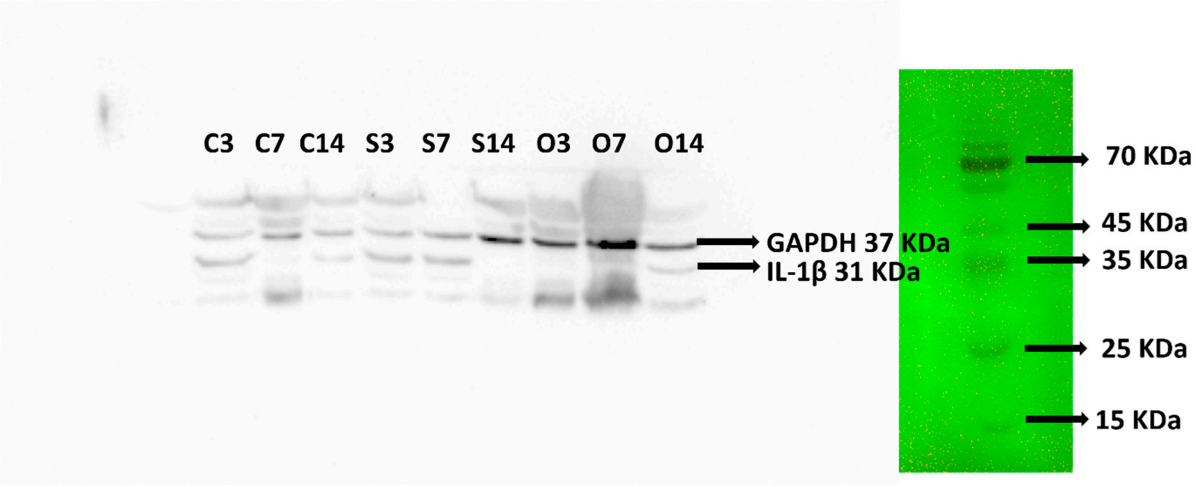

Figure S1. Full-length Western blot membrane showing intracellular pro-IL-1β (31 kDa) and its matching GAPDH (37 kDa) internal loading control across chronological wound tissue subsets.

Lane Descriptions: C3, C7, C14: Healthy Control Group tissue samples collected on Days 3, 7, and 14, respectively. **S3, S7, S14:** Untreated Streptozotocin (STZ)-induced Diabetic Group tissue samples collected on Days 3, 7, and 14, respectively. **O3, O7, O14:** STZ + ONX-0914 Treated Diabetic Group tissue samples collected on Days 3, 7, and 14, respectively.

Right Panel: **(Visible Light):** Displays the standard pre-stained protein molecular weight ladder (labeled in kDa), verifying proper protein resolution, horizontal alignment, and successful electrotransfer onto the membrane.

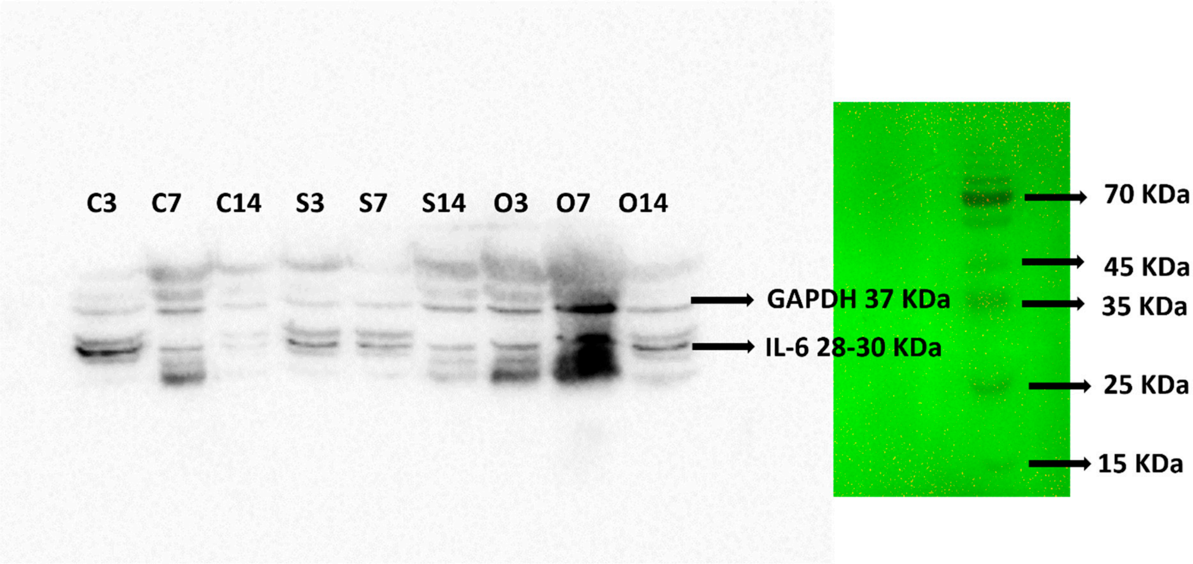

Figure S2. Full-length Western blot membrane showing IL-6 (28-30 kDa) expression and its matching GAPDH (37 kDa) internal loading control across chronological wound tissue subsets.

Lane Descriptions: C3, C7, C14: Healthy Control Group tissue samples collected on Days 3, 7, and 14, respectively. **S3, S7, S14:** Untreated Streptozotocin (STZ)-induced Diabetic Group tissue samples

collected on Days 3, 7, and 14, respectively. **O3, O7, O14:** STZ + ONX-0914 Treated Diabetic Group tissue samples collected on Days 3, 7, and 14, respectively.

Right Panel: **(Visible Light):** Displays the standard pre-stained protein molecular weight ladder (labeled in kDa), verifying proper protein resolution, horizontal alignment, and successful electrotransfer onto the membrane.

* **Note 1:** This membrane was stripped and reprobed following initial cytokine visualization to detect matching GAPDH loading controls sequentially on the same blot, as detailed in the Materials and Methods section of the main manuscript.

Note 2: Multiple lower bands or smeared patterns observed below the main target region represent different glycosylation states, physiological isoforms, or localized proteolytic degradation products of IL-6 common in actively remodeling diabetic wound microenvironments. **Only the specific, prominent target bands at approximately 28-30 kDa corresponding to mature IL-6 were utilized for quantitative densitometric normalization.**

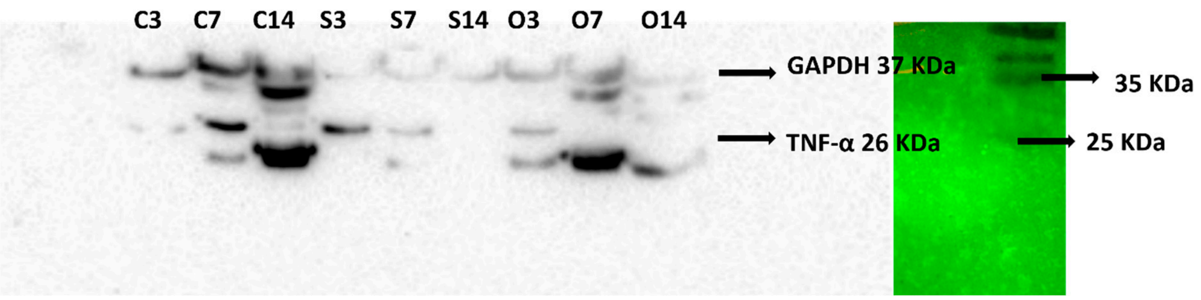

Figure S3. Full-length Western blot membrane showing intracellular pro-TNF-α (26 kDa) and its matching GAPDH (37 kDa) internal loading control across chronological wound tissue subsets.

Lane Descriptions: C3, C7, C14: Healthy Control Group tissue samples collected on Days 3, 7, and 14, respectively. **S3, S7, S14:** Untreated Streptozotocin (STZ)-induced Diabetic Group tissue samples collected on Days 3, 7, and 14, respectively. **O3, O7, O14:** STZ + ONX-0914 Treated Diabetic Group tissue samples collected on Days 3, 7, and 14, respectively. * **Note:** Only the specific bands at 26 kDa corresponding to pro-TNF-α were utilized for quantitative densitometric normalization; lower non-specific or degradation bands were strictly excluded from densitometry analysis.

Technical Note on Membrane Processing and Molecular Weight Verification: To maximize experimental efficiency and ensure high-affinity antibody hybridization for lower molecular weight proteins within the same run, the PVDF membrane was horizontally excised below the 40 kDa threshold following protein transfer.
